# The Role of Tracheostomy in Anaplastic Thyroid Carcinoma

**DOI:** 10.14740/wjon899w

**Published:** 2015-02-14

**Authors:** Jia Xu, Zhen Liao, Jing-Jia Li, Xi-Fu Wu, Shi-Min Zhuang

**Affiliations:** a Department of Otolaryngology-Head & Neck Surgery, The Third People Hospital of Dong Guan, Guangdong, 540600, China; b State Key Laboratory of Oncology in South China, Collaborative Innovation Center of Cancer Medicine Guangzhou, China; c Department of Operation Theater Services, Sun Yat-Sen University Cancer Center, Guangzhou, China; d Department of Otolaryngology-Head & Neck Surgery, The Third Affiliated Hospital of Sun Yat-sen University, 600 Tianhe Road, Guangzhou, Guangdong, 510630, China; e These authors contributed equally to this work

**Keywords:** Anaplastic thyroid carcinoma, Airway management, Tracheotomy

## Abstract

Even though management of thyroid cancer is generally standardized and has an overall excellent long-term outcome, anaplastic thyroid cancer (ATC) continues to be a major diagnostic and therapeutic challenge. ATC is an uncommon thyroid malignancy with a poor prognosis. American Thyroid Association guidelines acknowledge the complexity of airway management in these patients. We studied the literature with the aim of providing guidance in airway management in ATC. Tracheotomy can facilitate completion of palliative treatment in those patients with ATC and stridor. Given the short life expectancy of these patients, a balanced decision must be made regarding the role and timing of tracheotomy.

## Introduction

Anaplastic thyroid carcinoma (ATC) represents 2-5% of all thyroid malignancies and has the poorest prognosis [1]. It usually presents with a rapidly enlarging neck mass but most lethal of thyroid cancers [2, 3]. It is distressing that even after half a century of active investigation we have not made substantial progress in the management of this unique neoplasm. Whether this is related to the many stages of differentiation of ATC, or the inability to separate poorly differentiated and ATC, remains unclear at this time. The classic giant and spindle cell ATC grows rapidly and the average survival in this group of patients is between 3 and 6 months. Thyroid cancer is a spectrum of diseases. On one end of the spectrum, there is papillary thyroid carcinoma, one of the more indolent malignancies with a survival rate of more than 98%. At the other end of the spectrum is ATC, one of the most dreaded cancers, with a mortality rate of 98% [4]. The reason for the differences in biological behavior remains unclear. Whether there is a genetic progression from one end of the spectrum to the other remains to be seen. Local invasion from this mass is most often demonstrated by symptoms of hoarseness, stridor, and dyspnea. Airway compromise often occurs at an early stage secondary to external compression or invasion of the trachea [5]. Despite some changes in the management of ATC over recent years, such as the use of combined modality treatment, there has been limited progress with regards to length of patient survival. American Thyroid Association (ATA) guidelines have recently been published to assist clinicians in the management of ATC. These guidelines advocate that where the disease is considered to be resectable, surgery should be performed as initial management [6]. Overall ATC patients have a median survival of 5 months and less than 20% survive 1 year [2]. For the majority of these patients, the cause of death is airway compromise, or metastatic disease [7, 8]. Airway compromise may be apparent on initial presentation or may develop during treatment. Management of the airway in these patients is a challenge, particularly in relation to when, or indeed whether to tracheotomies, or surgically decompress these patients in a disease which carries such a poor prognosis. There has been limited literature concerning the complex issue of airway management in patients with ATCs up until the recently published ATA guidelines. These guidelines state that tracheotomies should only be performed in patients with severe airway distress and where possible this should take place in theater under a general anesthetic [6].

## Discussion

ATC is an aggressive disease, which as demonstrated the management of ATCs with airway invasion remains controversial. There is currently no standardized treatment capable of altering long-term survival [1, 2, 4-8]. The patient may have a compromised airway, ranging from mild to severe, or airway problems may develop and progress during the course of treatment, particularly during radiotherapy. An acute airway emergency may be precipitated after any manipulation, especially intubation, surgical intervention or even attempted percutaneous endoscopic gastrostomy. Some authors have described the use of endotracheal and endobronchial stents and laser vaporization of intraluminal tumor. These techniques have very limited value for ATC and are unlikely to avoid an acute airway catastrophe. Airway-related problems may be caused by extrinsic pressure on the trachea or central compartment of the neck from a large tumor, tracheal invasion by tumor, or paralyzed vocal cords on one or both sides. Paralysis of one cord with impending paralysis of the other side, bilateral vocal cord paralysis, tracheal invasion by the tumor or bleeding inside the trachea most often lead to acute airway distress. Approximately 20-30% of patients may present with acute airway problems with mild to moderate stridor. Their airway problems may be accentuated on exertion or minor upper airway infection which may lead to serious compromise of the airway. In the management of ATC, airway protection is a critical issue which requires considerable critical thinking and consideration of the quality of life, poor prognosis and limited treatment choices. Conservative treatment such as high humidification and steroids may be of some help temporarily but unlikely to help as the disease progresses. From the time of diagnosis of ATC, these issues need to be discussed by the multidisciplinary group as to how to maintain and manage the airway should the patient develop acute airway distress. The issues related to emergency intubation need to be discussed well in advance with the patient and the family.

The airway issues may be related to direct infiltration of the tumor in the tracheal lumen. Patients may initially present with airway distress and stridor, in which one vocal cord may be completely paralyzed and sluggish movement found in the opposite vocal cord, or laryngeal edema caused by compression of the structures in the central compartment of the neck. Occasionally, locally aggressive thyroid cancer may infiltrate submucosally or into the lumen of the trachea, at which time the airway may be considerably compromised, or the patient may bleed in the tracheal lumen, leading to acute airway distress and rapid fatality. Patients may develop acute airway distress during the course of radiation therapy because of a compromised airway and laryngeal edema. Airway management is quite complex in these patients and should be addressed, depending on the patient’s and the family’s wishes, by the availability of definitive treatments and the extent of disease. It is also important to secure the airway in these patients until diagnosis of lymphoma is ruled out and diagnosis of ATC is confirmed. Patients with lymphoma can expect complete improvement in their airway.

Tracheostomy procedures can be quite difficult because there may be extensive tumor in front of the trachea, making it practically impossible to identify the trachea or tracheal lumen. It is also difficult to secure a tracheostomy tube in such patients because of the distance between the skin and the trachea. The trachea is a posterior mediastinal structure, and there may be a considerable distance, especially when filled with tumor, between the skin and the tracheostomy site. Under these circumstances, the best approach would be a cricothyrotomy. Generally, a higher midline incision would be of help to expose the thyroid cartilage and search for the cricothyroid membrane. Occasionally, a partial tumoral resection may be necessary for better exposure of the cricothyroid area. A long tracheostomy tube is generally recommended to bypass tracheal narrowing. Occasionally, the tracheal narrowing and compression may extend almost up to carina because of extensive disease involving the mediastinum. Under these circumstances, maintaining the airway may be very difficult. Some patients may require positive pressure ventilation to keep the airway open.

Occasionally, airway management and airway intervention with tracheostomy may become a philosophical matter for patients with incurable, advanced, and universally fatal disease. This requires intense discussion with the patient and the family members, and the harsh realities of management of ATC need to be discussed. The patient or the family may not wish any airway intervention attempted. Invariably, the patient will require hospitalization or nursing care after the tracheostomy. The patient may have numerous issues related to excessive secretions. Swallowing may also be a problem, and patients may require a feeding tube or long-term gastrostomy.

Even though airway management is a difficult problem in the treatment of ATC, very little has been published in the literature. Shaha et al described acute airway distress in patients with thyroid pathology [9]. Similarly, extensive experience was reported by Gaissert et al from Massachusetts General Hospital, in which experience was described for tracheal resection and segmental laryngotracheal resection for invasive thyroid carcinoma [10]. Holting et al described their experience of tracheostomy for emergency respiratory management for ATC [11]. The authors reported that survival was significantly lower in the group of patients requiring tracheostomy. This is quite likely related to the advanced nature of the disease in patients requiring tracheostomy. However, because of patients with ATC a majority are more likely to suffer because of tracheostomy. The same authors reported problems with tracheostomy related to locally invasive ATC [12]. They reported worse survival rates in patients undergoing tracheostomy for ATC and that external radiation therapy often was delayed or could not be administered because of local complications from the tracheostomy. Overall, management of the airway in patients presenting with ATC continues to be a major challenge, and the treating physicians should be familiar with the issues related to tracheostomy and cricothyrotomy, and decisions should be made in conjunction with the patient’s and family’s wishes.

## Conclusion

Management of the airway in ATC is always a controversial area of debate. Airway intervention is dangerous and many ask, even after successful tracheotomy is it worth the negative quality of life implications. Patients with acute airway symptoms may require tracheotomy, and in those circumstances a cricothyroidotomy is advised for rapid airway entry. These cases, in the ATC setting require experienced head and neck surgeons and carry a high mortality rate. It may also be of assistance to first insert a rigid bronchoscope to maintain ventilation. Orientate the beveled end of the bronchoscope anteriorly such that the light of the scope may assist the operating surgeon locating the trachea. A marginal survival benefit may be appreciated with immediate tracheotomy; however, the quality of life drops with copious volumes of mucus, blood and tumor causing considerable patient discomfort and amounts to meaningless prolongation of life. In these circumstances palliative care physicians are essential for their specialty advice and leadership.

## References

[1] Ain KB (1999). Anaplastic thyroid carcinoma: a therapeutic challenge. Semin Surg Oncol.

[2] Smallridge RC, Copland JA (2010). Anaplastic thyroid carcinoma: pathogenesis and emerging therapies. Clin Oncol (R Coll Radiol).

[3] Smallridge RC (2012). Approach to the patient with anaplastic thyroid carcinoma. J Clin Endocrinol Metab.

[4] Shaha AR, Ferlito A, Owen RP, Silver CE, Rodrigo JP, Haigentz M, Mendenhall WM (2013). Airway issues in anaplastic thyroid carcinoma. Eur Arch Otorhinolaryngol.

[5] Wein RO, Weber RS (2011). Anaplastic thyroid carcinoma: palliation or treatment?. Curr Opin Otolaryngol Head Neck Surg.

[6] Smallridge RC, Ain KB, Asa SL, Bible KC, Brierley JD, Burman KD, Kebebew E (2012). American Thyroid Association guidelines for management of patients with anaplastic thyroid cancer. Thyroid.

[7] Lang BH, Lo CY (2007). Surgical options in undifferentiated thyroid carcinoma. World J Surg.

[8] Shaha AR (2008). Airway management in anaplastic thyroid carcinoma. Laryngoscope.

[9] Shaha A, Alfonso A, Jaffe BM (1987). Acute airway distress due to thyroid pathology. Surgery.

[10] Gaissert HA, Honings J, Grillo HC, Donahue DM, Wain JC, Wright CD, Mathisen DJ (2007). Segmental laryngotracheal and tracheal resection for invasive thyroid carcinoma. Ann Thorac Surg.

[11] Holting T, Meybier H, Buhr H (1989). [Problems of tracheotomy in locally invasive anaplastic thyroid cancer]. Langenbecks Arch Chir.

[12] Holting T, Meybier H, Buhr H (1990). [Status of tracheotomy in treatment of the respiratory emergency in anaplastic thyroid cancer]. Wien Klin Wochenschr.

